# Adapting SHIVs *In Vivo* Selects for Envelope-Mediated Interferon-α Resistance

**DOI:** 10.1371/journal.ppat.1005727

**Published:** 2016-07-11

**Authors:** David F. Boyd, Amit Sharma, Daryl Humes, Cecilia Cheng-Mayer, Julie Overbaugh

**Affiliations:** 1 Division of Human Biology, Fred Hutchinson Cancer Research Center, Seattle, Washington, United States of America; 2 Pathobiology Graduate Program, University of Washington, Seattle, Washington, United States of America; 3 Aaron Diamond Research Center, New York, New York, United States of America; Emory University, UNITED STATES

## Abstract

Lentiviruses are able to establish persistent infection in their respective hosts despite a potent type-I interferon (IFN-I) response following transmission. A number of IFN-I-induced host factors that are able to inhibit lentiviral replication *in vitro* have been identified, and these studies suggest a role for IFN-induced factors as barriers to cross-species transmission. However, the ability of these factors to inhibit viral replication *in vivo* has not been well characterized, nor have the viral determinants that contribute to evasion or antagonism of the host IFN-I response. In this study, we hypothesized that the host IFN-I response serves as a strong selective pressure in the context of SIV/HIV chimeric virus (SHIV) infection of macaques and sought to identify the viral determinants that contribute to IFN-I resistance. We assessed the ability of SHIVs encoding HIV-1 sequences adapted by serial passage in macaques versus SHIVs encoding HIV sequences isolated directly from infected individuals to replicate in the presence of IFNα in macaque lymphocytes. We demonstrate that passage in macaques selects for IFNα resistant viruses that have higher replication kinetics and increased envelope content. SHIVs that encode HIV-1 sequences derived directly from infected humans were sensitive to IFNα –induced inhibition whereas SHIVs obtained after passage in macaques were not. This evolutionary process was directly observed in viruses that were serially passaged during the first few months of infection–a time when the IFNα response is high. Differences in IFNα sensitivity mapped to HIV-1 envelope and were associated with increased envelope levels despite similar mRNA expression, suggesting a post-transcriptional mechanism. These studies highlight critical differences in IFNα sensitivity between HIV-1 sequences in infected people and those used in SHIV models.

## Author Summary

The innate immune system is an important host defense against viral infection. Recently, there has been significant interest in characterizing the innate immune response to HIV-1 infection, in particular the role of type-I interferon (IFN-I). Understanding the interaction of HIV-1 with the innate immune system is particularly important for the development of animal models of infection as innate host factors present potential species-specific barriers to the establishment of persistent infection. One of the most commonly used animal models of HIV-1 infection is chimeric SIV/HIV (SHIV) infection of macaques. Here, we demonstrate that the process of adapting SHIVs for replication in macaques selects for viruses that are resistant to the IFNα response, and we identity important viral determinants that contribute to this resistance. This improved understanding of virus interactions with the innate immune response may facilitate the development of improved animal models of HIV-1 infection.

## Introduction

The innate immune system presents the first host defense against viral infection. Host cells are able to sense the presence of viral infection and respond by producing type-I interferon (IFN-I), which, in turn, leads to the up-regulation of hundreds of host genes that are potentially antiviral [1,2]. Infection with HIV-1 in people and SIV in non-human primates induces a robust IFN-I response within days of infection [3–7]. IFN-I production, including IFNα, is part of a larger systemic cytokine storm that precedes the establishment of set-point viral load suggesting that the IFN-I response may play a role in limiting viral replication during acute infection and influence disease progression [8]. In support of this hypothesis, a recent study of SIV infection in rhesus macaques demonstrated that blocking the IFN-I response resulted in higher plasma viral loads during acute infection, increased reservoir size and faster progression to AIDS [9].

Despite the presence of a robust antiviral IFN-I response to infection, lentiviruses are able to replicate to high levels during acute infection and establish persistent infection in their hosts. Some recent studies have provided evidence that the innate immune response selects for HIV-1 variants that are relatively resistant to IFN-I during transmission [10,11]. The biological properties that contribute to the ability of some HIV-1 variants to resist the IFN-I response remain unclear.

One possible explanation for differences in IFN-I sensitivity of HIV-1 variants is that they have different abilities to evade or antagonize downstream effectors of the IFN-I response. Over the last decade, host antiviral proteins, referred to as restriction factors, have been identified that act at multiple stages of the lentiviral life cycle and directly inhibit viral replication [8,12]. Many of the restriction factors are induced by IFN-I [8,12]. Because the IFN-I-induced factors are effective at inhibiting viral replication, lentiviruses have evolved mechanisms to evade or antagonize their activity. Indeed, the human orthologs of the IFN-I-induced restriction factors that inhibit HIV-1 replication are largely inactive against HIV-1 because of the specificity of the viral antagonist for the human protein. The mechanisms of restriction factor inhibition and viral antagonism and the importance of these interactions for establishing productive infection *in vitro* have been carefully elucidated. However, the role of the IFN-I response in limiting viral replication and mechanisms of viral evasion/antagonism in the context of infection *in vivo* is less clear. Relevant to this, HIV-specific restriction factors have been largely studied for their ability to inhibit HIV-1 variants derived after passage in cell culture and less is known about the IFN-I-induced responses that inhibit viruses replicating in infected individuals.

Due to the selective pressure of restriction factors, lentiviral proteins are adapted to antagonize factors in their respective hosts and often act in a species-specific manner [8,13]. For example, HIV-1 proteins are able to antagonize human restriction factors but are unable to effectively counteract the non-human primate orthologs. For this reason, SIV/HIV chimeric viruses (SHIVs) used to study HIV-1 pathogenesis in macaques encode SIV antagonists of well-characterized macaque restriction factors. The HIV-1 genes encoded in SHIVs typically include the *env* gene that encodes the Envelope surface glycoprotein (Env). In most cases, SHIVs require multiple rounds of adaptation in lab-culture and/or by animal-animal serial passage in macaques in order to increase replication capacity and pathogenicity [14]. Often the process of animal-animal serial passage is performed within the first two weeks of infection when levels of IFN-I are highest in the animals [4,5,15–17], providing a possible selective pressure to drive changes in the virus. Thus, SHIVs that have been adapted in macaques present a unique opportunity to study the mechanism of adaptation to IFN-I response.

The goals of this study were to determine whether the process of adapting SHIVs for increased replication capacity and pathogenicity in macaques selects for variants that are resistant to the host’s IFN-I response and to identify the biological changes in the virus that contribute to IFN-I resistance. Given the fact that the majority of SHIVs studied to date encode HIV-1 variants derived from cell culture and represent the select group of SHIVs that were able to infect macaques, we also asked whether there are differences in IFN-I sensitivity of these viruses compared to SHIVs encoding HIV-1 sequences isolated directly from infected individuals. We demonstrate that envelope differences selected *in vivo* allow SHIVs to adapt to the IFNα response; adapted HIV-1 variants encode IFNα resistant Envs, whereas Envs obtained directly from infected individuals early in their infection are sensitive, suggesting that IFNα may have an inhibitory effect on viruses spreading in humans that has not been observed through the study of adapted viruses. These findings suggest that Env plays an important role in evading or antagonizing the IFNα response. Identification of IFNα resistant HIV-1 Env variants may facilitate the development of challenge viruses for macaque models of HIV-1 infection.

## Results

### Assessment of IFNα sensitivity of SHIVs by replication time course

In order to test the hypothesis that adaptation of SHIVs results in IFN-I resistance, we compared a panel of nine SHIVs for their ability to replicate in macaque cells in the presence or absence of IFNα. The panel of SHIVs included four viruses that encode HIV-1 sequences isolated directly from infected individuals (MG505, Q23, QF495, and BG505) with the latter three from early infection [18–20], two viruses that encode HIV-1 sequences obtained from individuals during late-stage chronic infection and adapted in lab-culture in human cells (AD8 and SF162) [21,22] and three viruses that encode HIV-1 sequences adapted by animal-animal passage in macaques (AD8-EO, SF162P3 and 1157ipd3N4), two of which represent the animal-passaged derivatives of the lab-adapted viruses [16,21,23] (S1 Table). Most HIV-1 variants circulating in people are unable to use the macaque CD4 receptor for entry into cells [24], therefore, SHIVs that were made from HIV-1 variants isolated directly from individuals encode single amino acid changes that allow them to use the macaque CD4 receptor for entry. HIV-1 variants encoding these changes are able to use the macaque CD4 for entry at levels similar to those of adapted SHIVs [24], and the viruses chosen for study represent those that were infectious in macaque CD4+ T cells. Otherwise, the HIV-1 sequences of these SHIVs are unmodified from the sequences found in the infected individual and will be referred to as circulating SHIVs as they are representative of HIV-1 variants circulating in human populations.

**Fig 1:**
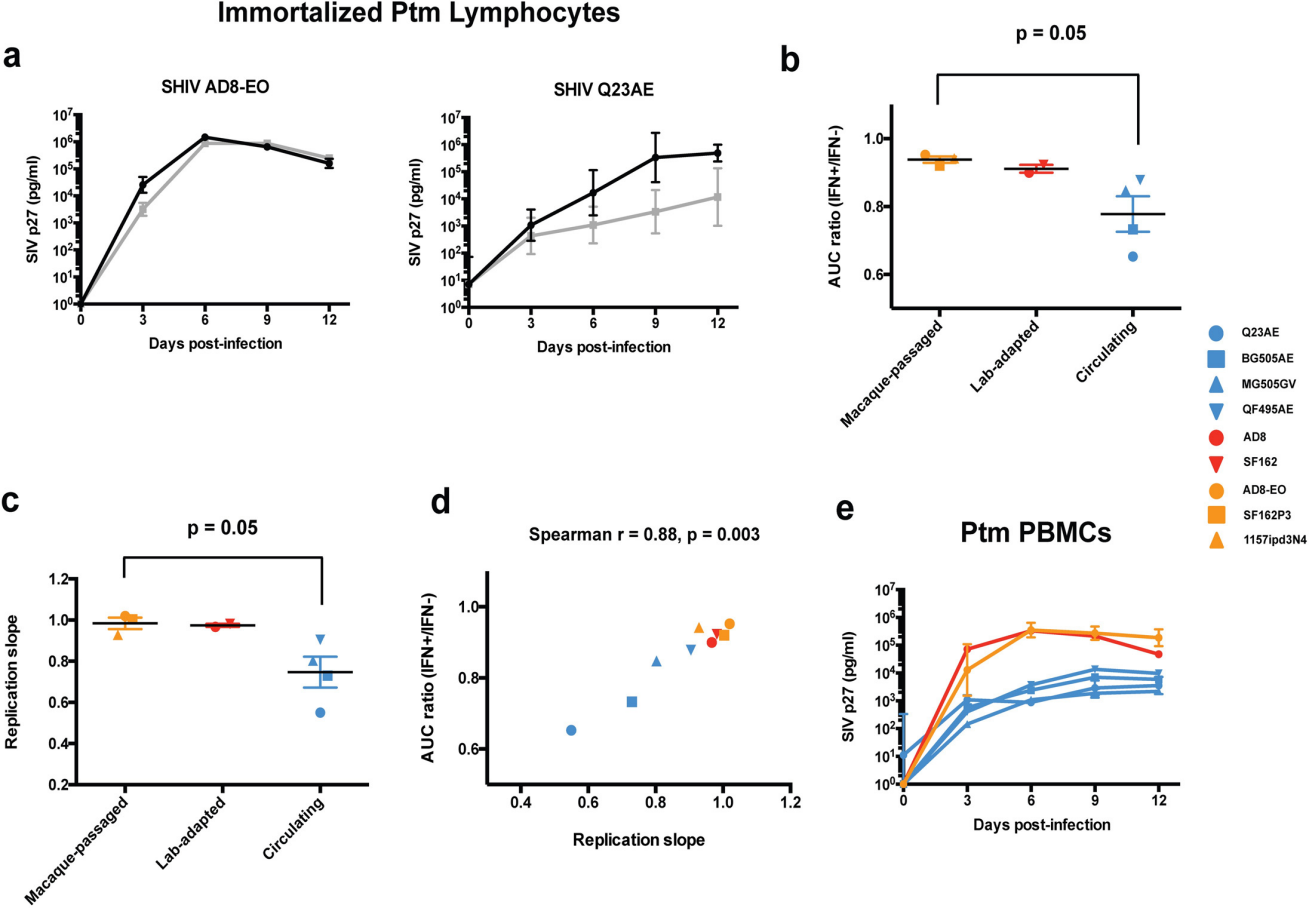
Effect of IFNα treatment on viral replication kinetics of SHIVs in pig-tailed macaque cells. (a) The ability of each SHIV to replicate in the presence of 1000 U/ml of IFNα-2a (gray lines) or absence of treatment (black lines) was assessed in immortalized Ptm lymphocytes. Replication curves for the remaining seven SHIVs are presented in S1 Fig. The identity of each SHIV is indicated above the chart. SIV p27 concentration in infected cell supernatants is plotted vs. days post-infection. Data points represent the average of at least two independent experiments and error bars indicate SD. (b) Comparison of IFN-I sensitivity of SHIVs in immortalized Ptm lymphocytes. The area-under-curve ratio (IFN+/IFN-) is plotted for macaque-passaged (orange), lab-cultured (red) and circulating (blue) SHIVs. Each data point, which is shown with a unique symbol for each virus, represents the average of at least two independent experiments. Error bars represent SEM. A two-tailed student’s t-test was used to compare AUC ratios of macaque-passaged vs. circulating SHIVs. (c) The replication slope in immortalized Ptm lymphocytes for each SHIV is presented. Error bars indicate the SEM. Mean replication slopes of macaque-passaged and circulating SHIVs were compared by two-tailed student’s t test. (d) Comparison of replication kinetics and IFNα sensitivity in immortalized Ptm lymphocytes. The relationship between the AUC ratio (IFN+/IFN-) and the replication slope across the panel of nine SHIVs is presented. Each dot represents a SHIV variant. Spearman r represents the Spearman rank correlation coefficient. (e) Replication curves for macaque-passaged, lab-cultured and circulating SHIVs in primary Ptm PBMCs. Data points represent the average of two independent experiments, and error bars indicate SD.

We assessed the ability of the panel of SHIVs to replicate in immortalized pig-tailed macaque (Ptm) CD4+ lymphocytes [25] in the presence and absence of IFNα-2a at a concentration similar to that observed in natural infection (1000 U/ml) [4,5] (Fig 1A, S1 Fig). Intracellular staining for two IFNα-stimulated proteins, MX1 and IFIT1, showed that nearly all immortalized Ptm lymphocytes responded to IFNα treatment (S2 Fig). IFNα sensitivity was measured as the ratio of the area-under curve (AUC) of the replication curve in the IFN-treated cells to the AUC of the replication curve in the untreated cells. For example, SHIV AD8-EO, a pathogenic, macaque-passaged virus, replicated in the presence of IFNα nearly as well as the untreated cells (Fig 1A) and had an AUC ratio (IFN+/IFN-) of 0.96. In contrast, SHIV Q23AE, a circulating SHIV, exhibited a pronounced IFNα-induced inhibition of viral replication corresponding to an ~100-fold reduction in SIV p27 levels at nine dpi and had an AUC ratio of 0.68 (Fig 1A). The other seven viruses exhibited a range of inhibition between these two ends of the spectrum (S1 Fig). We observed the same patterns of IFNα-induced inhibition when we pre-treated the cells with IFNα-2a at 24 hours prior to infection (S3 Fig). Overall, SHIVs adapted by macaque-passage and by lab-culture exhibited higher AUC ratios than circulating SHIVs indicating resistance to IFNα treatment (Fig 1B). Comparing AUC ratios, macaque-passaged SHIVs were significantly more resistant to IFNα treatment compared to circulating SHIVs (0.94 vs. 0.78, p = 0.05).

### Association between viral replication kinetics and IFNα sensitivity

The replication kinetics of the nine SHIVs were defined using the data from the replication time course studies where replication differences between viruses were evident even in the absence of IFNα. For example, SHIV AD8-EO demonstrated rapid replication kinetics in untreated cells reaching peak virus levels of >10^6^ pg/ml of SIV p27 by six dpi (Fig 1A). In contrast, SHIV Q23AE reached lower peak virus levels of >10^5^ pg/ml of SIV p27 at nine dpi. In order to compare replication kinetics across the panel of nine SHIVs, we determined a summary measure of viral replication based on the slope of a best-fit straight line of the replication curve in untreated immortalized Ptm lymphocytes during the first six days of infection. Comparing replication slopes, macaque-passaged SHIVs replicated faster than circulating SHIVs (0.98 vs. 0.75, p = 0.05), and lab-adapted SHIVs were more similar to the animal-adapted SHIVs (Fig 1C). There was a strong positive correlation between replication kinetics and IFNα resistance as measured by the AUC ratio (Spearman r = 0.88, p = 0.003) (Fig 1D). Representative macaque-passaged and lab-cultured SHIVs also exhibited faster replication than circulating SHIVs in primary Ptm PBMCs (Fig 1E). Thus, macaque-passaged SHIVs exhibited higher replication kinetics and greater IFNα resistance compared to the circulating SHIVs, and the replication kinetics in untreated immortalized Ptm lymphocytes correlate with the ability of the virus to overcome the IFNα response.

### HIV-1 Env determines replication capacity and sensitivity to IFNα

**Fig 2:**
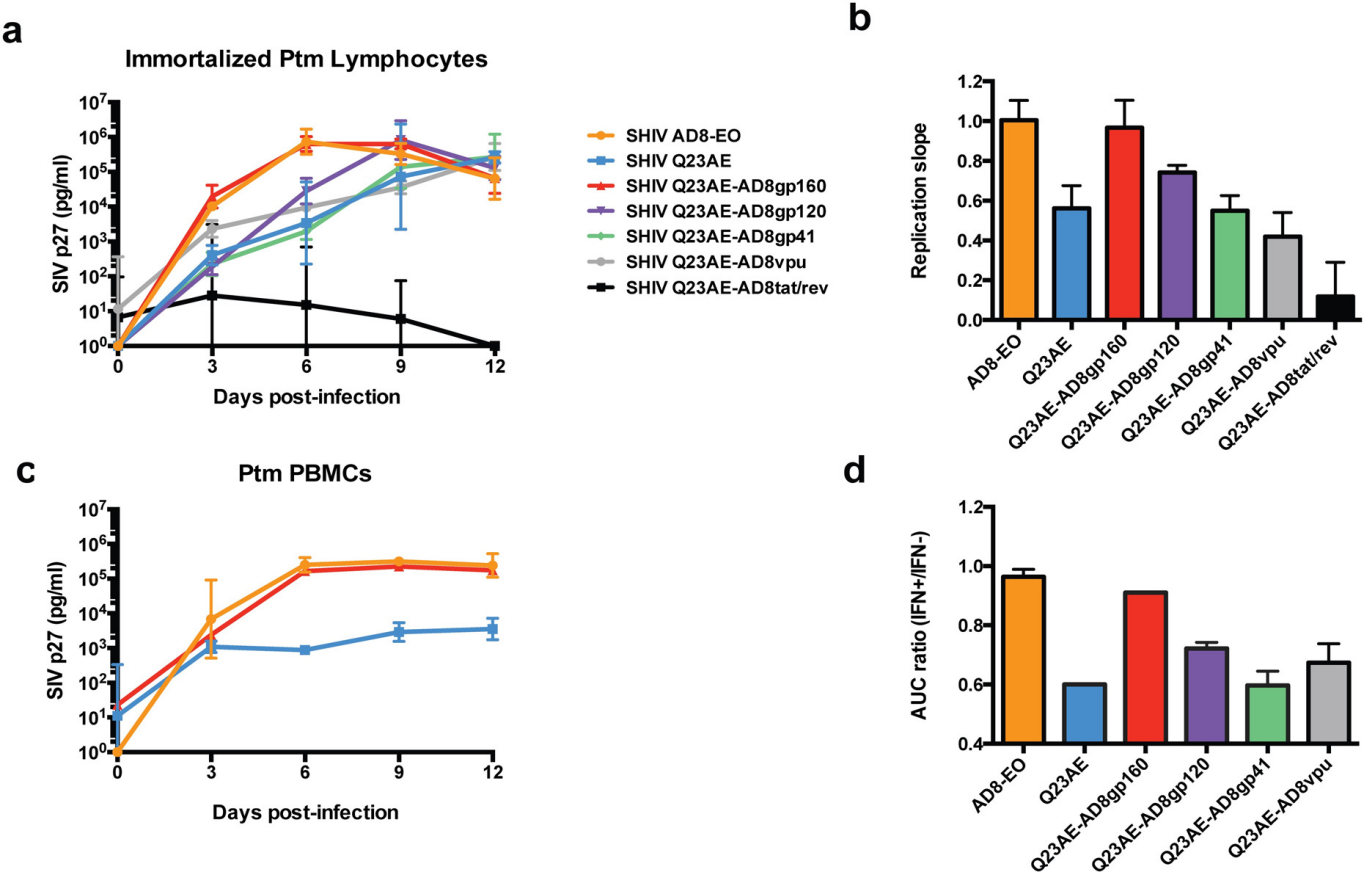
Analysis of chimeric SHIVs in Ptm cells. (a) Replication kinetics of SHIV AD8-EO, SHIV Q23AE and chimeras (Q23AE-AD8gp160, Q23AE-gp120 and Q23AE-gp41, Q23AE-AD8vpu and Q23AE-AD8tat/rev) were determined by measuring SIV p27 levels over a 12-day time course in immortalized Ptm lymphocytes. The key at the right indicates the color corresponding to each SHIV tested. Data points represent the average of two independent experiments, and error bars represent SD. (b) The slope of the replication curve in the first six days of infection immortalized Ptm lymphocytes of all chimeras generated were compared to those of SHIV AD8-EO and SHIV Q23AE. Bars represent the average replication slope of at least two independent experiments. (c) Replication kinetics of SHIV Q23AE-AD8gp160, SHIV AD8-EO and SHIV Q23AE in primary Ptm PBMCs. (d) Comparison of the area-under-curve (AUC) ratio among SHIV chimeras. AUC ratio was determined by dividing the AUC of the replication curve in IFNα treated cells by the AUC in untreated cells. AUC ratio (IFN+/IFN-) is plotted on the y-axis for each of the indicated chimeras. Data represent the average of at least two independent experiments, and error bars indicate the SD.

In order to identify the viral determinant(s) of replication capacity and IFNα sensitivity, we generated chimeras between the viruses that exhibited the greatest difference in replication kinetics and IFNα sensitivity, SHIV AD8-EO, which is a prototype animal-adapted SHIV, and SHIV Q23AE, which represents a circulating SHIV. Because SHIV Q23AE was generated by cloning full HIV-1 *vpu* and *env* genes directly into SHIV AD8-EO [21], the SHIV Q23AE and SHIV AD8-EO are isogenic except for the HIV-1 genes *vpu*, *env* and the second exons of *tat/rev*; thus, biological differences between them must be due to the HIV-1 sequences. Introduction of the entire *env* gene from SHIV AD8-EO to SHIV Q23AE resulted in complete recovery of replication capacity (Fig 2A). Introduction of the gp120 surface subunit of Env resulted in a modest increase in replication kinetics while introduction of the gp41 trans-membrane subunit did not result in any detectable increase in replication (Fig 2A and 2B). Because regions of the *env* gene contain overlapping reading frames with *vpu* and *tat/rev*, we also introduced the full *vpu* gene and the second *tat* exon, including a portion of *rev*, from SHIV AD8-EO to SHIV Q23AE. Introduction of neither *vpu* nor the second *tat/rev* exon resulted in a significant increase in replication kinetics (Fig 2B). Introduction of the full *env* gene from SHIV AD8-EO to SHIV Q23AE also restored high-level replication capacity in primary Ptm PBMCs where differences in replication are similar to those of the immortalized Ptm lymphocytes (Fig 2C).

The chimeras were then examined for their ability to replicate in the presence of IFNα treatment. Introduction of the entire HIV-1 *env* gene from SHIV AD8-EO to SHIV Q23AE resulted in a nearly complete recovery of IFNα resistance (Fig 2D). The gp120 chimera exhibited a modest increase in IFNα resistance while neither the gp41 nor vpu chimera demonstrated any detectable increase in IFNα resistance. Because of the poor replication capacity of the tat/rev chimera, we were unable to determine its IFNα sensitivity using the AUC ratio. Thus, the HIV-1 *env* gene is a major determinant of replication and of resistance to the IFNα response in Ptm cells.

### Virion-associated HIV-1 Env content contributes to replication kinetics and IFNα resistance

**Fig 3:**
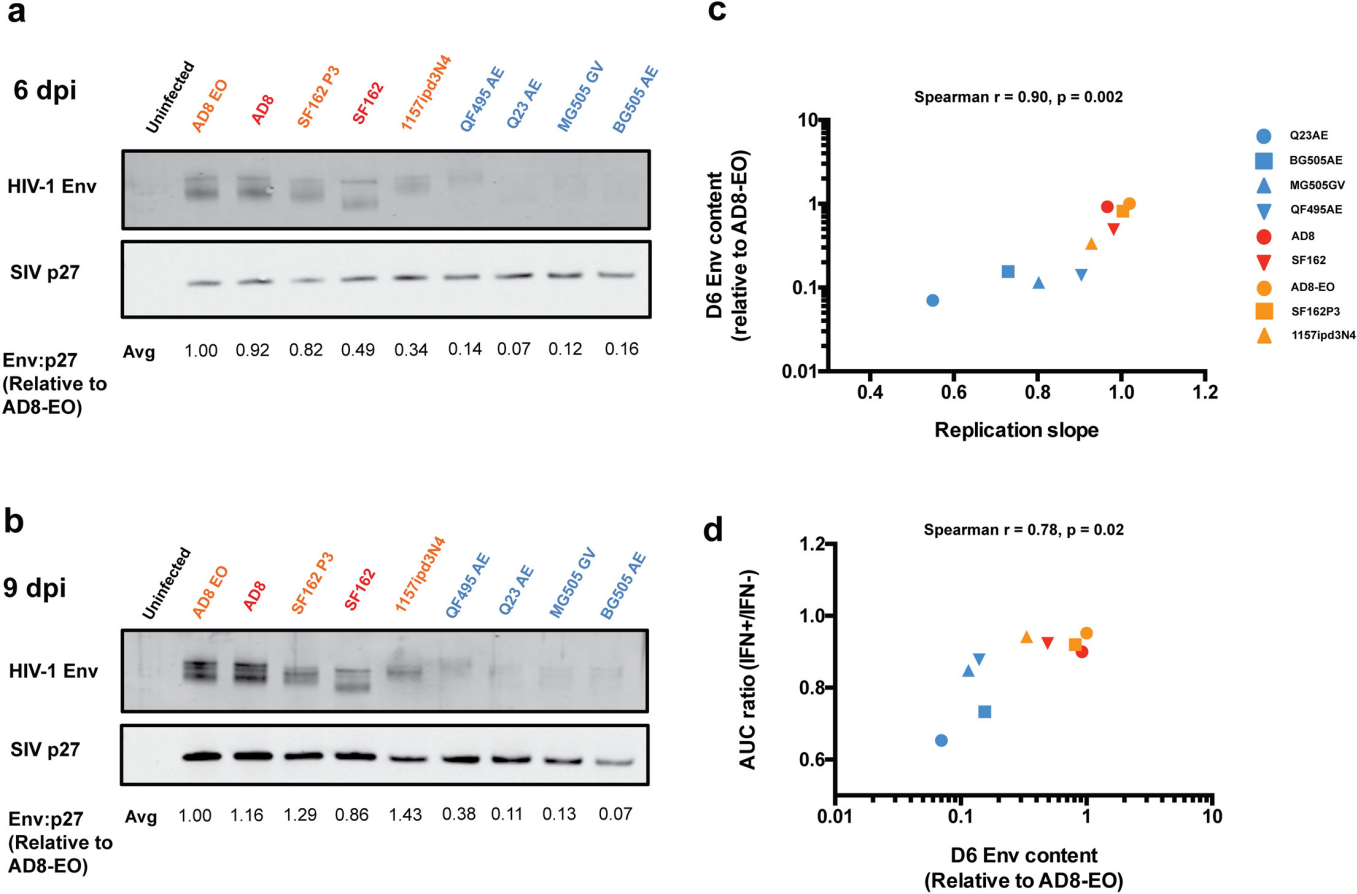
Western blot analysis of virion-associated Env content in cell-free supernatants harvested at six (a) and nine (b) days post-infection. The name of each SHIV being tested is indicated above each lane. Color-coding indicates whether the SHIVs has been adapted by macaque-passage (orange), in lab-culture (red), or represents a circulating HIV-1 variant (blue). For each sample, 5 ng of SIV p27 was loaded into each lane. The Env:p27 signal relative to SHIV AD8-EO indicated at the bottom of the panel for each virus is the average for two independent experiments. (c) Correlation between the slope of viral replication in the first 6 days of infection and the average virion-associated HIV-1 Env content of cell-free virions from 6 dpi was determined. (d) Correlation between average virion-associated Env content at 6 dpi and IFNα sensitivity as measured by AUC ratio (IFN+/IFN-). Spearman r represents the Spearman rank correlation coefficient.

To address the basis for the effect of Env on replication capacity, we measured the amount of Env protein present in virions harvested from infected immortalized Ptm lymphocytes for the panel of nine SHIVs. For the day six viral lysates, the levels of HIV-1 Env in virus expressed from cells infected with the SHIVs adapted in lab-culture or by animal-passage were consistently higher than that of virus from cells infected with the four circulating SHIVs (Fig 3A). These differences are exemplified by SHIV AD8-EO and SHIV Q23AE where there was a >10-fold difference between HIV-1 Env relative to SIV Gag p27. These patterns of Env content were similar at nine dpi (Fig 3B). In the purified virion lysates, we did not observe evidence of contamination from infected cells, for example the presence of unprocessed Gag, although we cannot definitively rule out very low levels of infected cell debris. Because the panel of SHIVs encodes HIV-1 Envs from diverse subtypes, we probed for Env using two different primary antibodies, a polyclonal rabbit sera from animals immunized with a subtype A Env protein [26] and HIVIG, antibodies pooled from HIV-1+ patients (NIH AIDS Reagent Program). We observed the same patterns of Env content using either of the primary antibodies indicating that differences in HIV-1 Env detection were not due to differences in antibody recognition of the diverse proteins (S4 Fig).

In addition to virion-associated Env content, we determined the relative infectivity of prototype macaque-passaged (AD8-EO) and circulating (Q23AE) SHIVs by measuring TCID_50_ in immortalized Ptm lymphocytes (S5 Fig). We found that the TCID50 values were very similar between the two viruses and in each case about 100-fold lower than the titer defined using TZM-bl cells. Thus, while the input levels of infectious virus were lower based on the TCID50 assay (MOI of 0.0002 rather than 0.02), the infecting virus titer was similar between the two viruses in our experiments. Interestingly, when we normalized TCID50 to p27 levels, we found that the infectivity of SHIV AD8-EO was approximately 30-fold higher than SHIV Q23AE (S5 Fig) suggesting that SHIVAD8-EO may have a higher ratio of infectious to non-infectious particles than SHIV Q23AE. Thus, the approach of using virus titer rather than p27 levels provided the best approach to normalizing infectious virus input (S5 Fig).

We next compared virion-associated Env content to replication kinetics across all nine viruses from Fig 3A in immortalized Ptm lymphocytes. We observed a strong positive correlation between the replication slope and Env content of virions produced at six dpi (Spearman r = 0.90, p = 0.002) (Fig 3C). Overall, SHIVs adapted by macaque-passage and in lab-culture had higher virion Env content and higher replication kinetics compared to circulating SHIVs.

Considering our previous finding that replication kinetics positively correlated with the ability to overcome the IFNα response, we compared virion-associated Env content and AUC ratio (IFN+/IFN-). We observed a positive correlation between Env content in virions and resistance to IFNα treatment (Spearman r = 0.78, p = 0.02) suggesting that Env content is linked to the ability to overcome the IFNα response (Fig 3D).

### Levels of HIV-1 Env expression in SHIV-infected cells

**Fig 4:**
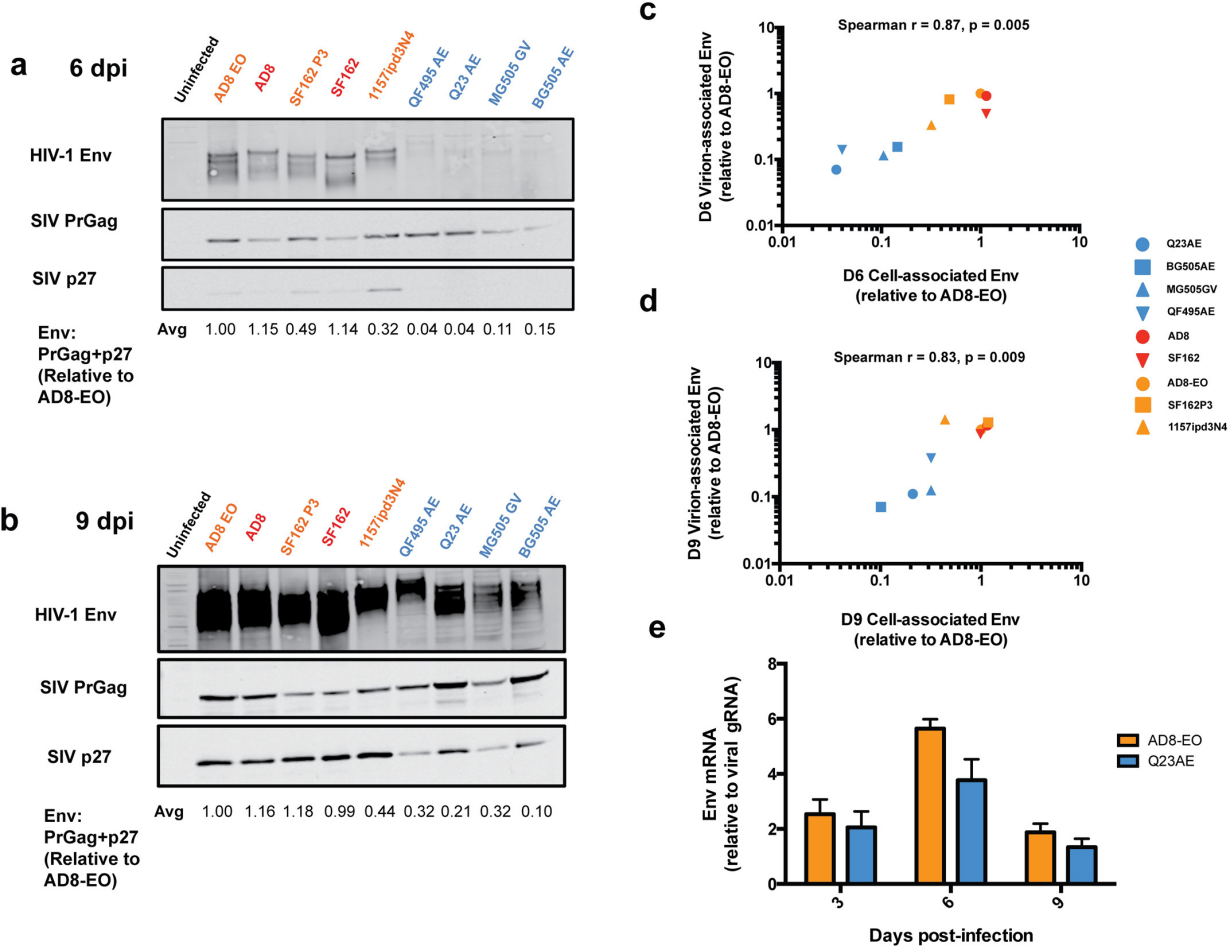
Analysis of cell-associated HIV-1 Env expression in SHIV-infected immortalized Ptm lymphocytes. SHIV-infected Ptm cells harvested and lysed at six (a) and nine (b) days post-infection analyzed for HIV-1 Env expression. The values below the bottom panel indicate the average ratio of HIV-1 Env signal to the sum of precursor Gag (PrGag) and p27 (Env: PrGag + p27) for two independent experiments. Color-coding indicates whether the SHIVs has been adapted by macaque-passage (orange), in lab-culture (red), or represents a circulating HIV-1 variant (blue). (c,d) Correlation between virion-associated Env, plotted on the y-axis, and cell-associated Env expression at 6 dpi (c) and 9 dpi (d). Spearman r represents the Spearman rank correlation coefficient as determined by Prism Graphpad Version 6.0c. (e) Comparison Env mRNA levels relative to un-spliced viral genomic RNA (gRNA) levels at three, six and nine dpi between SHIV AD8-EO (orange) and SHIV Q23AE (blue). Ratio of Env mRNA relative to viral gRNA is presented on the y-axis. The bars represent the average of two independent experiments and error bars represent the SD.

The observed differences in Env content in SHIV virions could be due to variation in synthesis within the infected cells or to variation in incorporation into newly formed virions. In order to address these two possibilities, we measured the amount of HIV-1 Env expressed in SHIV-infected immortalized Ptm lymphocytes. The pattern of Env detection was similar in cells as in virions: at six dpi, there was higher steady state Env levels in cells infected with SHIVs adapted by macaque-passage or in lab-culture compared to circulating SHIVs despite detection of PrGag at comparable levels (Fig 4A), and the same was true at nine dpi (Fig 4B). For example, SHIV AD8-EO had >30-fold (1.0 vs. 0.04) more HIV-1 Env relative to total Gag compared to SHIV Q23AE. For the nine SHIVs tested, there was a strong positive correlation between the relative steady state intracellular Env expression levels and relative virion-associated Env content at both six dpi (Spearman r = 0.87, p = 0.005) (Fig 4C) and nine dpi (Spearman r = 0.83, p = 0.009) (Fig 4D) suggesting that differences in Env content in virions are reflective of differences in Env levels in the infected cells.

To address whether differences in intracellular HIV-1 Env expression are the result of variation in transcription and splicing of *env* mRNA, we measured spliced *env* mRNA and un-spliced viral genomic RNA by reverse transcriptase quantitative PCR (RT-qPCR) for SHIV AD8-EO and SHIV Q23AE. At three and nine dpi, we did not observe any difference between SHIV AD8-EO and SHIV Q23AE with respect to the amount of spliced *env* mRNA relative to un-spliced viral genomic RNA (Fig 4E), although there was a statistically significant difference at six dpi; SHIV AD8-EO had ~1.5-fold more spliced *env* mRNA compared to SHIV Q23AE (5.6 vs. 3.8, p = 0.04). Given the small magnitude of this RNA difference compared to protein differences (>30-fold) and that differences were not observed at other time points where protein differences were observed, these findings suggest that low levels of intracellular HIV-1 Env expression are due to post-transcriptional events in SHIV-infected Ptm lymphocytes.

### IFNα sensitivity of SHIVs adapted *in vivo*

**Fig 5:**
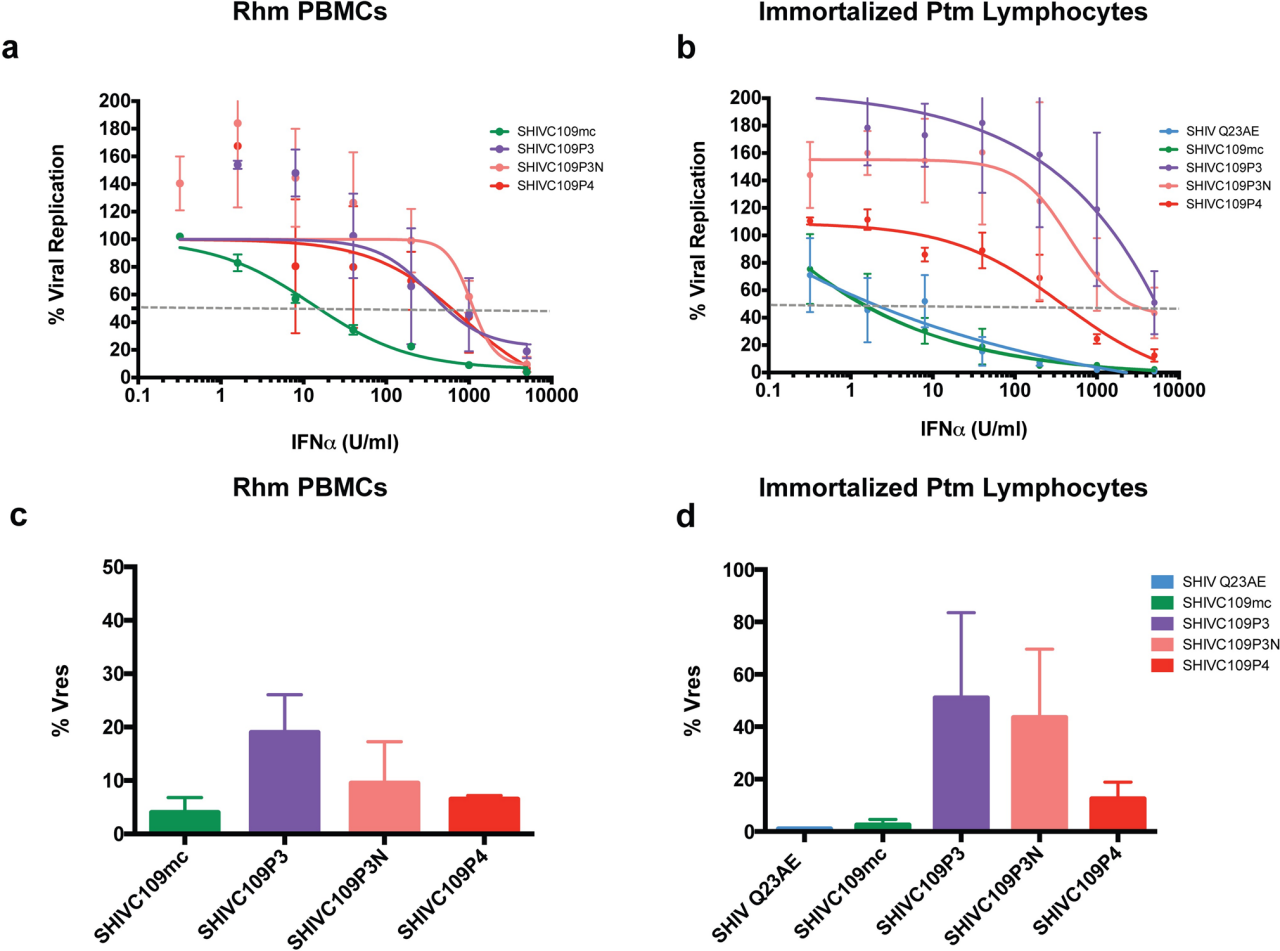
Assessment of IFNα sensitivity by IFN IC_50_ assay. Dose-dependent inhibition of viral replication by IFNα. Viral replication as a percentage of replication in untreated Rhm PBMCs (a) or immortalized Ptm lymphocytes (b) is plotted vs. concentration of IFNα-2a (U/ml). Data for the un-adapted, parental clone (SHIVC109mc) and macaque-passaged isolates (SHIVC109P3, SHIVC109P3N and SHIVC109P4) are presented. SHIV Q23AE serves as a positive control for IFN-induced inhibition. Each data point represents the average of two independent experiments. Data are fitted to a non-linear curve using Prism Graphpad version 6.0c. The dashed grey line indicates 50% inhibition of viral replication used to determine the IC_50_ value. Comparison of % residual viral replication (Vres) at the highest concentration of IFNα-2a (5000 U/ml) between SHIVC109 isolates in RhmPBMCs (c) and immortalized Ptm lymphocytes (d). Data represent the average of two independent experiments, and error bars represent the SD.

The finding that animal-passaged SHIVs were the most IFNα resistant of all viruses tested suggested that *in vivo* adaptation results in increased resistance to the IFNα response. To test the hypothesis that the process of adapting SHIVs by serial animal-animal passage in macaques increases IFNα resistance of SHIVs, we examined a collection of related SHIVs derived from a parental SHIV encoding an HIV-1 Env variant obtained without culturing, similar to the other circulating SHIVs described above [17,27]. We tested the IFNα sensitivity of the parental molecular clone (SHIVC109mc), two isolates from the third animal passage–one from early (SHIVC109P3) and one from late in infection (SHIVC109P3N)–and an isolate from the fourth animal passage (SHIVC109P4). For these studies, we applied an assay that allowed us to determined the amount of IFNα-2a required to inhibit 50% of viral replication (IFNα IC_50_) [11] as a more quantitative method to assess IFN sensitivity. Because these viruses were adapted for replication by serial passage in rhesus macaques, we first tested their IFNα sensitivity in primary Rhm PBMCs (Fig 5A). The parental circulating SHIVC109mc was the most sensitive to IFNα (12.8 U/ml), with adapted SHIVs derived from passage of this virus showing 25–80-fold increased resistance. The three passaged viruses were generally similar in their sensitivity to IFNα to each other (340–1,000 U/ml), suggesting that adaptation to become resistant to IFNα occurred by the time of the third passage.

Very similar results were observed in immortalized Ptm lymphocytes. The parental molecular clone SHIVC109mc was highly sensitive to IFNα treatment (IFNα IC_50_ 1.6 U/ml), similar to SHIV Q23AE (Fig 5B). Each of the macaque-passaged isolates was more resistant to the IFNα response induced in macaque lymphocytes. Both of the isolates from passage three exhibited IFNα IC_50_ values >5,000 U/ml while the passage four isolate was moderately sensitive (IFNα IC_50_ 330 U/ml). The % Vres values, which measures residual virus replication at the highest IFN level tested, demonstrated similar patterns of IFNα resistance (Fig 5C and 5D). Replication of the parental virus was nearly completely inhibited at the highest concentration of IFNα while the passage three isolates demonstrated higher residual replication. This result was consistent between both Rhm PBMCs and immortalized Ptm lymphocytes although overall residual replication was higher for the passaged isolates in Ptm cells. Interestingly, several SHIVs that were resistant to IFNα exhibited increased replication in the presence of IFNα. This increase in replication could be the result of proliferation of cells that were initially protected from infection at early time points but later became infected.

**Fig 6:**
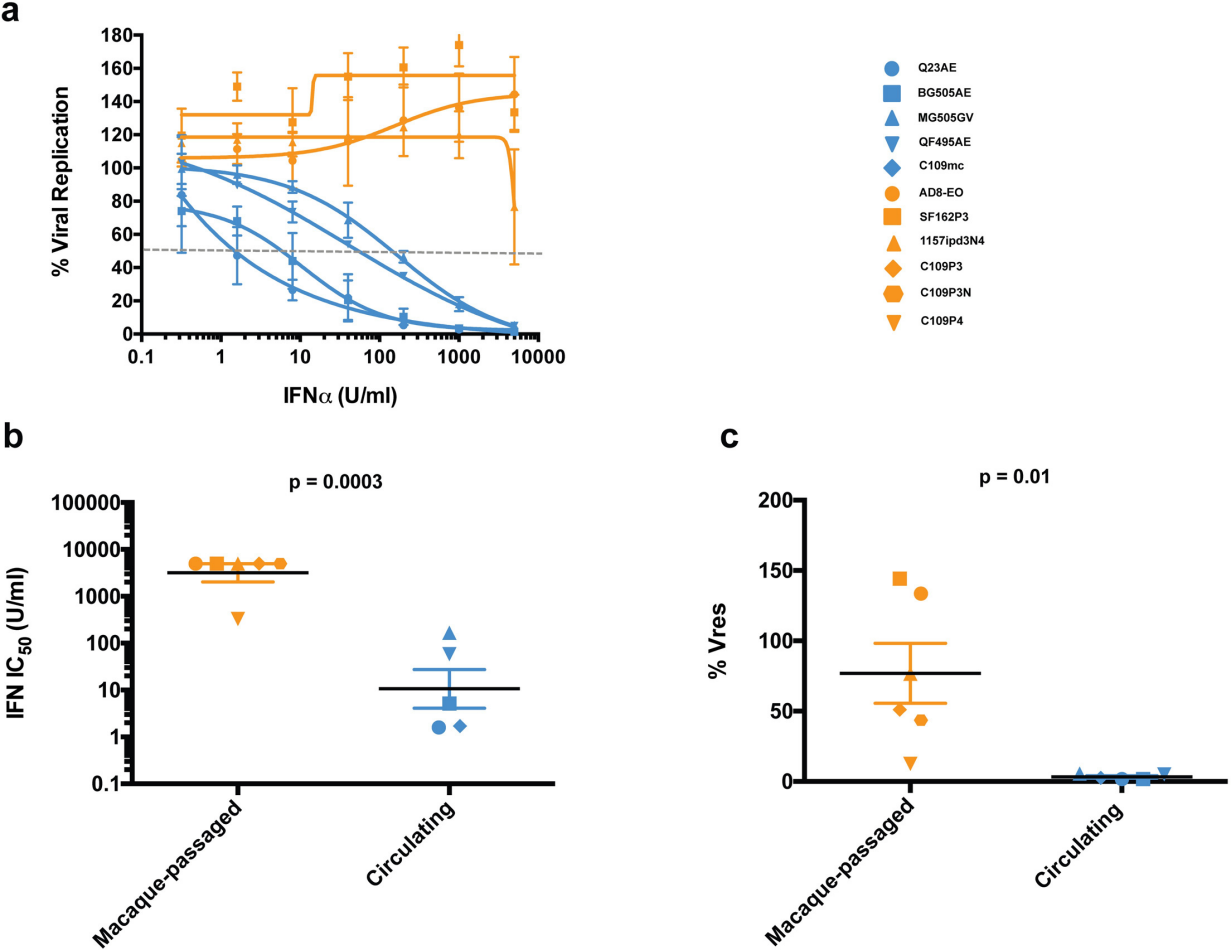
Comparison of IFNα sensitivity of macaque-passaged and circulating SHIVs. (a) Dose-dependent inhibition of viral replication by IFNα. Each data point represents the average of least two independent experiments, and error bas represent the SD. The identity of each SHIV is indicated at the right. (b) Comparison of IFN IC_50_ values between macaque-passaged and circulating SHIVs. Each data point represents the average IC_50_ value for one SHIV variant, and error bars represent SEM. IC_50_ values were log-transformed, and mean IC_50_ values were compared by two-tailed student’s t test. (c) Comparison of % residual viral replication (Vres) at the highest concentration of IFNα-2a (5000 U/ml) between macaque-passaged and circulating SHIVs. Each data point represents the average IC_50_ value for one SHIV variant, and error bars represent SEM. Mean % Vres values were compared by two-tailed student’s t test.

In order to sample a larger collection of viruses and compare these measures of IFNα sensitivity between circulating and animal adapted viruses, we defined IFNα IC50 and % Vres values for the three animal-adapted and four circulating SHIVs examined at a single IFN concentration in Fig 1. The seven viruses exhibited a range of IFNα IC_50_ values (1.7–5,000 U/ml). For example, SHIV Q23AE was highly sensitive to IFNα treatment and exhibited a dose-dependent inhibition of viral replication with an IC_50_ value of 1.7 U/ml (Fig 6A). In contrast, SHIV AD8-EO was completely IFNα resistant and did not exhibit inhibition of viral replication at any of the concentrations; the other animal-passaged SHIVs were similarly IFNα resistant.

The data from these SHIVs allowed us to compare the IFNα sensitivity of SHIVs encoding Envs isolated directly from people and macaque-passaged SHIVs from a total of 11 viruses. The macaque-passaged SHIVs (n = 6) were significantly more resistant to IFNα compared to the circulating SHIVs (n = 5) with respect to both IFNα IC_50_ value (3,180 vs. 10.6 U/ml, p = 0.0003) and % Vres (76.9 vs. 3.3%, p = 0.01) (Fig 6B and 6C).

## Discussion

We took advantage of the SHIV macaque model to define the role of IFNα in infection and found that infection selects for viruses that are resistant to the inhibitory effects of IFNα in macaques. Pathogenic SHIVs, which have been developed to model HIV-1 transmission and pathogenesis in macaques, are resistant to IFNα, whereas the SHIVs based on HIV-1 variants circulating in humans, including transmitted viruses, are inhibited by IFNα. Differences in sensitivity to IFNα were determined by the HIV-1 Envelope protein, which is considered a key feature of the SHIV models. Our findings underscore critical differences between SHIVs adapted for replication in macaques and HIV-1 variants isolated directly from infected individuals, including those that were recently transmitted, which represent the most biologically relevant targets of HIV-1 vaccine and prevention efforts.

Resistance to IFNα inhibition was associated with higher replication capacity, which resulted from the process of adapting virus in animals. Both increased replication and resistance to IFNα were observed in three SHIVs derived from animal passage compared to their corresponding parental molecularly cloned viruses. In all three cases, the process of adapting SHIVs in macaques, including during the critical window of early infection, led to increased replication and IFNα resistance. The differences were most striking when comparing virus derived from a SHIV constructed from HIV-1 sequences derived directly from an infected individual early in their infection [27] and pathogenic SHIVs derived from it by serial passage during the first few months of infection, which includes a time when the IFNα response is high [3–7]. Interestingly, the IFNα sensitivity of this panel of viruses mirrors those of the *in vivo* viral replication: the parental cloned virus, SHIVC109mc, was the most sensitive to IFNα treatment and demonstrated the lowest peak viremia. The viruses from the third animal passage (SHIVC109P3 and SHIVC109P3N) demonstrated the greatest resistance to IFNα and the highest peak viremia *in vivo*, 100–1,000-fold higher than the parental virus. The virus from the fourth animal passage SHIVC109P4 was intermediate in terms of IFNα sensitivity and peak viremia between the parental and passage three isolates. A variety of amino acid substitutions and deletions in variable (V1V2, V3 and V4) and constant (C3) regions of Env occurred during adaptation [17], and thus, there are many potential amino acid changes that could contribute to IFNα resistance. Importantly, selection for IFNα resistance was observed in both Rhm PBMCs and immortalized Ptm lymphocytes, suggesting a similar mechanism of IFNα inhibition in both macaques. Taken together with data showing that blocking the IFNα response early in infection results in faster progression to AIDS [9], these results suggest that the pathogenicity of adapted SHIVs may in part reflect their selection for IFNα resistance. These findings also suggest that macaque IFNα response *in vivo* exerts a strong selective pressure on SHIVs.

Two of the parental SHIVs examined here (ADA, SF162) were derived from HIV-1 sequences that were isolated during late-stage chronic infection by passaging the virus *in vitro* [28,29]. These viruses showed greater resistance to IFNα than SHIVs encoding sequences obtained directly from infected individuals early in infection. Conditions under which these viruses were derived in culture may have selected for IFNα resistance, perhaps by selecting those viruses with increased replication kinetics. It is also possible that these HIV-1 variants were already selected for features that made them less sensitive to IFNα inhibition because they were derived from later in infection. The IFNα sensitivity of the SHIVs that have not undergone either cell culture or animal adaptation to IFNα inhibition may help explain why it has been so difficult to identify SHIVs that encode HIV-1 variants isolated directly from infected individuals and are pathogenic in macaques. Indeed, our studies predict that the rare pathogenic SHIVs derived directly from HIV-1 infected people [30–32] may represent a small subset of variants that are resistant to IFNα inhibition, potentially allowing them to antagonize the early IFN storm and seed the viral reservoir to establish a persistent infection.

By generating chimeras between a pathogenic, macaque-passaged SHIV and a circulating SHIV encoding HIV-1 sequences isolated directly from an infected individual, we demonstrated that HIV-1 Env is a critical determinant of the ability of SHIVs to overcome the IFNα response. We found that the underlying mechanism for role of Env in IFNα sensitivity is related to Env protein levels. Interestingly, differences in Env protein levels were not reflected in *env* RNA levels. There was a ≤1.5 fold difference in spliced *env* RNA levels between adapted and circulating SHIVs compared to protein differences of >30-fold. These findings suggest that low levels of intracellular HIV-1 Env expression are due to post-transcriptional events in SHIV-infected macaque cells. While Env content correlated with IFNα sensitivity, we observed differences in the IFNα sensitivity of lab-cultured and macaque-passaged SHIVs despite similar levels of Env. Thus, while our results overall suggest that Env content plays an important role in the ability to overcome the macaque IFNα response, other determinants in the envelope protein may also contribute to IFNα resistance.

The finding that IFNα resistance mapped to HIV-1 *env* and was the result of high Env content was somewhat surprising considering that HIV-1 Env has not previously been implicated in viral antagonism of the IFN-I response, although some studies have suggested that IFN-I treatment predominantly affects very early stages of viral replication [33,34]. These findings raise several interesting possibilities. One is that HIV-1 Envs from adapted SHIVs act directly in evasion or antagonism of the IFNα response by protein-protein interactions with an IFN-induced host factor(s). An alternative hypothesis is that high HIV-1 Env expression/content contributes to increased kinetics allowing the virus to overcome the IFNα response by saturating IFN-induced factor(s). There is some precedent for this model as the ability to saturate IFN-induced restriction factors has been demonstrated *in vitro* [35–38]. In support of the model that an IFN-induced inhibitory factor is being saturated, we found a strong positive correlation between replication kinetics and IFNα resistance.

IFN–induced, HIV-specific restriction factors are typically species-specific, presumably because HIV-1 has adapted to its human host. Thus, we expect that the IFN-induced factor(s) that limit replication of circulating SHIVs, but not animal adapted SHIVs, may have similar species-specificity. However, we could not directly test whether this pathway is active in human cells with the viruses studied here because SHIVs replicate poorly in human cells due to other host restrictions. A similar correlation between replication capacity and IFNα resistance was recently reported for HIV-1 in human lymphocytes [39].

Differences in virion-associated Env content between SHIVs adapted by lab-culture and/or macaque-passage and those based on circulating variants were reflected by similar differences in Env levels in infected cells. Cells infected with adapted SHIVs had high Env levels whereas those infected with circulating SHIVs had low Env levels. Low Env content could help minimize immune recognition. For example, low Env content has been suggested to reduce antibody avidity [40]. Given that HIV-1 can spread as both cell-free and cell-associated virus [41], one intriguing possibility to explain our finding is that high Env expression in infected cells is leading to increased cell-cell transmission and saturation of IFN-α inhibition of cell-cell virus spread. Interestingly, the pattern of IFNα inhibition was the same whether cells were pre-treated with IFNα or treated just after infection (S3 Fig); even in cells pretreated with IFNα, there is a delay in the inhibition due to IFNα. This could be a result of an effect on cell-cell transmission that is only seen after the initial round of cell-free virus infection. Alternatively, it may indicate the IFN-induced factor is packaged into virions and inhibits later rounds of infection. Finally, a technical explanation for the observed delay in IFNα inhibition could be that the p27 ELISA used to determine virus levels is not sensitive enough to detect differences at very low levels of viral replication.

Overall, the results of this study demonstrate differences in the IFNα sensitivity between SHIVs used to model HIV-1 infection and HIV-1 variants circulating in infected people. They also suggest that the common focus on lab-adapted viral variants may have limited the ability to identify important mechanisms underlying the IFN-I-induced inhibition of HIV-1 variants circulating in people. The results uncover a key role for envelope in the process of adaptation of lentiviruses to the IFNα response and help explain why SHIVs generally do not cause pathogenic infections in macaques without adaptation. These findings may provide insight into the development of improved SHIV challenge viruses for non-human primate models of HIV-1 infection that currently serve as gatekeepers for HIV-1 vaccine and prevention studies.

## Materials and Methods

### Construction of full-length proviral clones

Full-length proviral SHIV clones encoding the region spanning the *vpu* and *env* open reading frames were generated using SHIV AD8-EO as a vector [21]. Expression plasmids encoding *vpu* and *env* open reading frames for Q23ENV.17 [18], BG505.W6M.ENV.B1 [20], MG505.W0M.ENV.H3 [20], and QF495.23M.ENV.A3 [19] A204E and G312V variants were amplified using primers designed to introduce an EcoRI site 5’ of the *vpu* start codon and a SalI site immediately 3’ of the *env* stop codon. The amplicons were then digested and ligated into the SHIV AD8-EO full-length proviral plasmid using EcoRI and SalI. Chimeras between SHIV AD8-EO and SHIV Q23AE were generated by overlap-extension PCR.

The following full-length proviral plasmids of the parental SHIVs were also used in this study: SHIV AD8 [21] and SHIV SF162 [22].

### Production of virus

Full-length, replication-competent virus was generated by transfecting 2x10^6^ HEK 293T cells (American Type Culture Collection, Manassas, VA) with 4 μg of proviral plasmid DNA and 12 μl of Fugene 6 transfection reagent (Roche). Virus was harvested 48 hours post-transfection, passed through a 0.2 μm sterile filter and concentrated ~10-fold using a 100 kDa molecular weight protein concentrator (Amicon).

Replication-competent stocks of SHIV SF162P3 [23], SHIV 1157ipd3N4 [16], SHIVC109mc, SHIVC109P3, SHIV109P3N and SHIVC109P4 [17] were generated by expanding the virus in immortalized Ptm lymphocytes [25]. For each virus, 2x10^6^ cells were infected at an initial multiplicity of infection (MOI) of ~0.02 by spinoculation at 1200 x g for 90 minutes at room temperature. After spinoculation, cells were washed 1x with 1 ml of Iscove’s modified Dulbecco’s medium (IMDM) supplemented with 10% heat-inactivated FCS, 2 mM L-glutamine, 100 U of penicillin/ml, 100 μg of streptomycin/ml and 100 U of interleukin-2/ml (Chiron) (complete IMDM), re-suspended in 2.4 ml of media and plated in a 6-well plate. Every three days, infected cells and cell supernatant were harvested and separated by pelleting at 650 x g for five minutes at room temperature.

Aliquots of replication-competent virus were stored at -80°C. The viral titer of each viral stock was determined by infecting TZM-bl cells and counting the number of blue cells at 48 hours post-infection after staining for β-galactosidase activity [20].

### Viral replication assays

Replication of SHIVs was assessed using immortalized Ptm CD4+ lymphocytes [25] maintained in complete IMDM. One million Ptm lymphocytes were infected at an MOI of 0.02 by spinoculation as described above. In some cultures, recombinant human IFNα-2a (PBL Assay Science, Piscataway, NJ) was added at a final concentration of 1,000 U/ml five hours after the initial infection. Every three days, 400 μl of each cell supernatant was replaced, including with IFNα-2a if appropriate. SIV p27 concentrations were determined using a SIV p27 antigen ELISA (ABL, Rockville, MD).

For some experiments Ptm or Rhm PBMCs were used: these cells were isolated from whole macaque blood (Washington National Primate Research Center, Seattle, WA) from two separate donors using 95% Lymphoprep (STEMCELL, Vancouver, BC). Isolated PBMCs were stimulated for three days prior to infection with IL-2 (20 U/ml) and concanavalin A (5 μg/ml) in RPMI 1640 medium supplemented with 20% FCS and 2 mM L-glutamine. Ptm or Rhm PBMCs from two donors were pooled immediately prior to infection, and 1x10^6^ PBMCs were spinoculated and maintained as described for immortalized Ptm lymphocytes.

### Western blot analysis of HIV-1 Envelope content and expression

The amount of HIV-1 envelope (Env) was determined by semi-quantitative western blot using the LICOR Odyssey system. For virion-associated Env content, supernatants from infected immortalized Ptm lymphocyte cultures were pelleted through a 25% sucrose cushion by ultracentrifugation for 90 minutes at 28,000 rpm. Virus pellets were lysed in 70 μl of radioimmunoprecipitation assay (RIPA) buffer for 10 minutes at room temperature. The concentration of SIV p27 antigen in the virus lysates was determined by ELISA, and virus lysate input was normalized to 5 ng of p27. Western blotting was performed as described previously [42] using rabbit polyclonal anti-HIV-1 Env sera [26] and mouse anti-SIV p27 monoclonal antibody (ABL, catalog no. 4323). Both gp160 and gp120 bands were included in the quantification of Env signal. For western blotting of whole cell lysates, SHIV-infected immortalized Ptm lymphocytes were pelleted by spinning at 650 x g for 5 minutes at room temperature. Cell pellets were washed with 1x PBS and then lysed in 100 μl of RIPA buffer for 10 minutes at room temperature. Western blotting was performed as described for virus lysates.

### HIV-1 Envelope reverse transcriptase quantitative PCR

HIV-1 RNA was measured by reverse transcriptase quantitative PCR (RT-qPCR). Total RNA was isolated from SHIV-infected immortalized Ptm cells using the miRNeasy Mini Kit (Qiagen). For each reaction, 50 ng of total RNA, measured by Nanodrop spectrophotometer was amplified using the Superscript III Platinum SYBR Green One-Step RT-qPCR kit with ROX (Invitrogen). Primers 5’- AGGGACTTGGCAAATGGATTGTAC-3’ and 5’ GTGTAATAGGCCATCTGCCTGCC-3’ were used to amplify gag from unspliced RNA. To amplify splice *vpu/env* mRNA, the forward primer 5’-AGGAACCAACCACGACGGAGTGCTC -3’, which binds upstream of the splice donor site in the 5’ LTR, and reverse primer 5’-CATTGCCACTGTCTTCTGCTCTTTC-3’, which binds downstream of the Vpu start codon, were used. To amplify macaque β-actin mRNA, primers 5’-CAACCGCGAGAAGATGACCCAGATCATG-3’ and 5’-AGGATGGCATGGGGGAGGGCATAC-3’ were used. Relative levels of HIV-1 *env* mRNA for each virus was determined using the following equation: 2^-ΔΔ^ = [(C_T_ *env*—C_T_ *beta actin*)]—[(C_T_ *genomic*—C_T_ *beta actin*)] [43].

### SHIV IFNα sensitivity assay

For each SHIV, 4.25x10^6^ Ptm or Rhm lymphocytes were infected at an MOI of 0.02 in a final volume of 1.4 ml of complete IMDM using spinoculation. Approximately 2.5x10^5^ infected cells in 200 μl of media were plated in each well of a 96-well plate containing 50 μl of media containing the indicated concentrations of IFNα-2a; experiments were performed in duplicate. Cell supernatants were harvested at 7 days post-infection (dpi) and used to determine the amount of virus using TZM-bl cells (NIH AIDS Reagent Program). For the data analysis, all values were plotted and statistical analyses performed using Prism version 6.0c (GraphPad Software). Percent viral replication was determined by dividing the amount of β-galactosidase activity in the IFNα treated sample by the untreated sample. The concentration of IFNα at which 50% viral inhibition was achieved was interpolated from a non-linear, best-fit curve. The amount residual viral replication at the highest concentration of IFNα (5000 U/ml) was also determined.

### TCID_50_ assay

TCID_50_ assay was used to determine the end-point dilution of the SHIV stocks at which infection is detected in 50% of the immortalized Ptm CD4+ lymphocyte culture replicates. Seven serial 4-fold dilutions of the SHIV stocks were prepared in triplicate in 96-well flat-bottomed tissue culture plates. Briefly, the SHIV stocks were diluted 1:12 in complete IMDM and 200 μl of diluted virus was transferred to the first well of a 96-well plate. Next, the virus was serially diluted by transferring 50 μl of the virus to the subsequent well containing 150 μl of complete IMDM. 2 x 10^5^ immortalized Ptm CD4+ lymphocytes, in 50 μl of complete IMDM, were seeded in each well. Every 4 days 125 μl of the culture supernatant was removed from every well and replaced with fresh 150 μl of complete IMDM. On day 12, 100 μl of the culture supernatant from each well was harvested and tested for SIV p27 using a SIV p27 antigen ELISA. TCID_50_ was calculated using the Spearman-Kaber method.

## Supporting Information

S1 Table Summary of characteristics of SHIVs used in this study. The table indicates the identify of each SHIV, the identity of the HIV-1 variant encoded in each virus, the time post-infection at which the HIV-1 variant was isolated, any adaptation that took place *in vitro* or *in vivo* and references.
(TIF)

S1 Fig Effect of IFNα treatment on viral replication kinetics of SHIVs in immortalized pig-tailed macaque lymphocytes. The ability of each SHIV to replicate in the presence of 1000 U/ml of IFNα-2a (gray lines) or absence of treatment (black lines) was assessed in immortalized Ptm lymphocytes. The identity of each SHIV is indicated above the chart. SIV p27 concentration in infected cell supernatants is plotted vs. days post-infection. Data points represent the average of at least two independent experiments and error bars indicate SD.
(TIF)

S2 Fig Induction of IFNα-stimulated genes in the macaque cells upon IFNα treatment. The immortalized pig-tailed macaque (Ptm) lymphocytes were left untreated or treated with 1000 U/ml of IFNα-2a for 24 hr. Cells were fixed, permeabilized and intracellular staining was performed for MX1 (a) and IFIT1 (b) followed by flow cytometric analysis. The histograms represent the expression of MX1 and IFIT1 as measured by fluorescein isothiocyanate (FITC)-conjugated secondary antibody in the untreated (red) or IFNα-2a-treated (blue) cells. Appropriate isotype control antibodies were used for intracellular staining of MX1 and IFIT1. The data are representative of two independent experiments.
(TIF)

S3 Fig Effect of IFNα pretreatment on viral replication kinetics of SHIVs in macaque cells. The ability of each SHIV to replicate in the presence of 1000 U/ml of IFNα-2a (gray lines) or absence of treatment (black lines) was assessed in immortalized pig-tailed macaque (Ptm) lymphocytes. Ptm cells were untreated (black square), only pre-treated 24 hr prior to infection with IFNα-2a (black circle), treated with IFNα-2a 5 hr post-infection (grey square), or both pre-treated and treated 5 hr post-infection with IFNα-2a (grey circle). The identity of each SHIV is indicated above the chart. SIV p27 concentration in infected cell supernatants is plotted vs. days post-infection. The data are representative of two independent experiments.
(TIF)

S4 Fig Comparison of HIV-1 envelope western blot using different primary antibodies. Five ng of SIV p27, as measured by ELISA, was loaded into each lane. The blot was probed with α-Env polyclonal rabbit sera [26] (top panel) and antibodies pooled from HIV-1+ patients (NIH AIDS Reagent Program) (bottom middle panel). The identity of the SHIV variant tested is indicated above each well, and SHIVs are color-coded as macaque-passaged (orange), lab-cultured (red) or circulating (blue) SHIVs.
(TIF)

S5 Fig Comparison of TZM-bl and TCID_50_ viral titers for SHIV AD8-EO and SHIV Q23AE. The number of infectious units (IU) per ml as determined by the TZM-bl assay and the viral titer as determined by TCID_50_ in immortalized Ptm lymphocytes are indicated. p27 represents the concentration of SIV p27 capsid protein in the viral stocks as determined by ELISA. At the right, the virus input to achieve an MOI of 0.02 for replication assays in macaque cells is indicated as number of infectious units or TCID_50._ In addition, the amount of SIV p27 added to the infections for each virus is indicated.
(TIF)
