# Supplementary figures and images for: Adapting SHIVs In Vivo Selects for Envelope-Mediated Interferon-α Resistance
Source: PLoS Pathog. 2016 Jul 11;12(7):e1005727. doi: 10.1371/journal.ppat.1005727 (PMC4939950; doi:10.1371/journal.ppat.1005727)

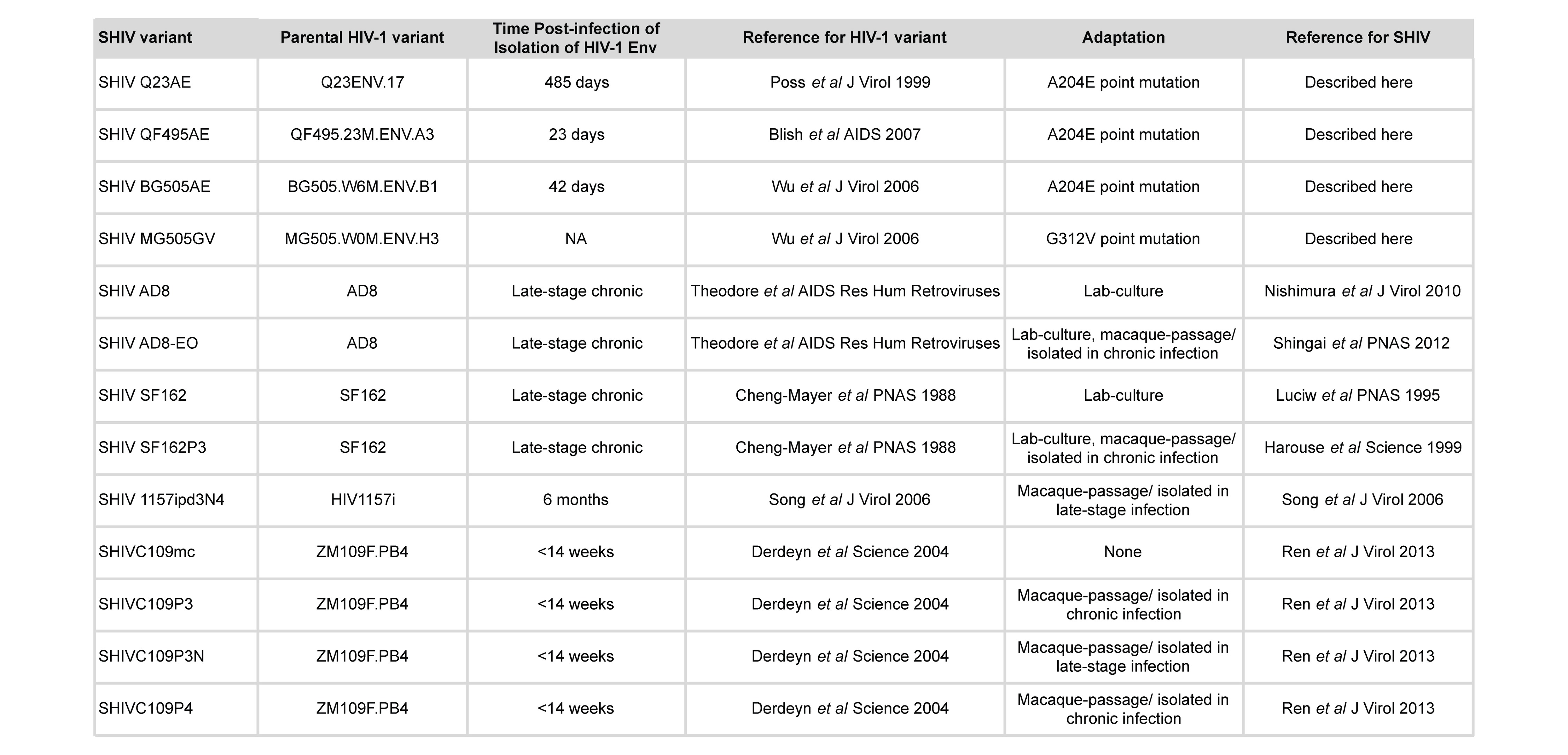

Supplement: S1 Table — The table indicates the identify of each SHIV, the identity of the HIV-1 variant encoded in each virus, the time post-infection at which the HIV-1 variant was isolated, any adaptation that took place in vitro or in vivo and references. (TIF) [file ppat.1005727.s001.tif]

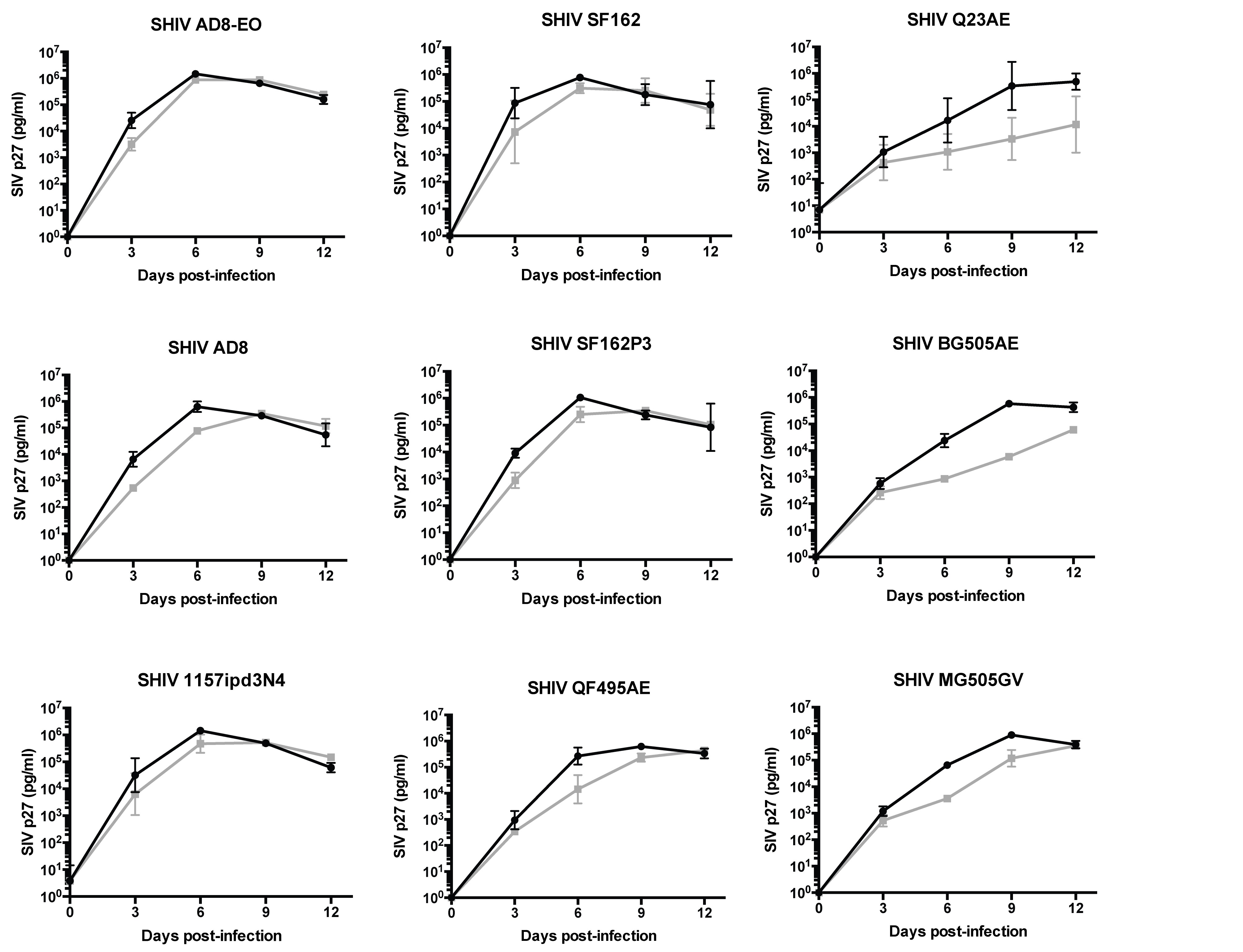

Supplement: S1 Fig — The ability of each SHIV to replicate in the presence of 1000 U/ml of IFNα-2a (gray lines) or absence of treatment (black lines) was assessed in immortalized Ptm lymphocytes. The identity of each SHIV is indicated above the chart. SIV p27 concentration in infected cell supernatants is plotted vs. days post-infection. Data points represent the average of at least two independent experiments and error bars indicate SD. (TIF) [file ppat.1005727.s002.tif]

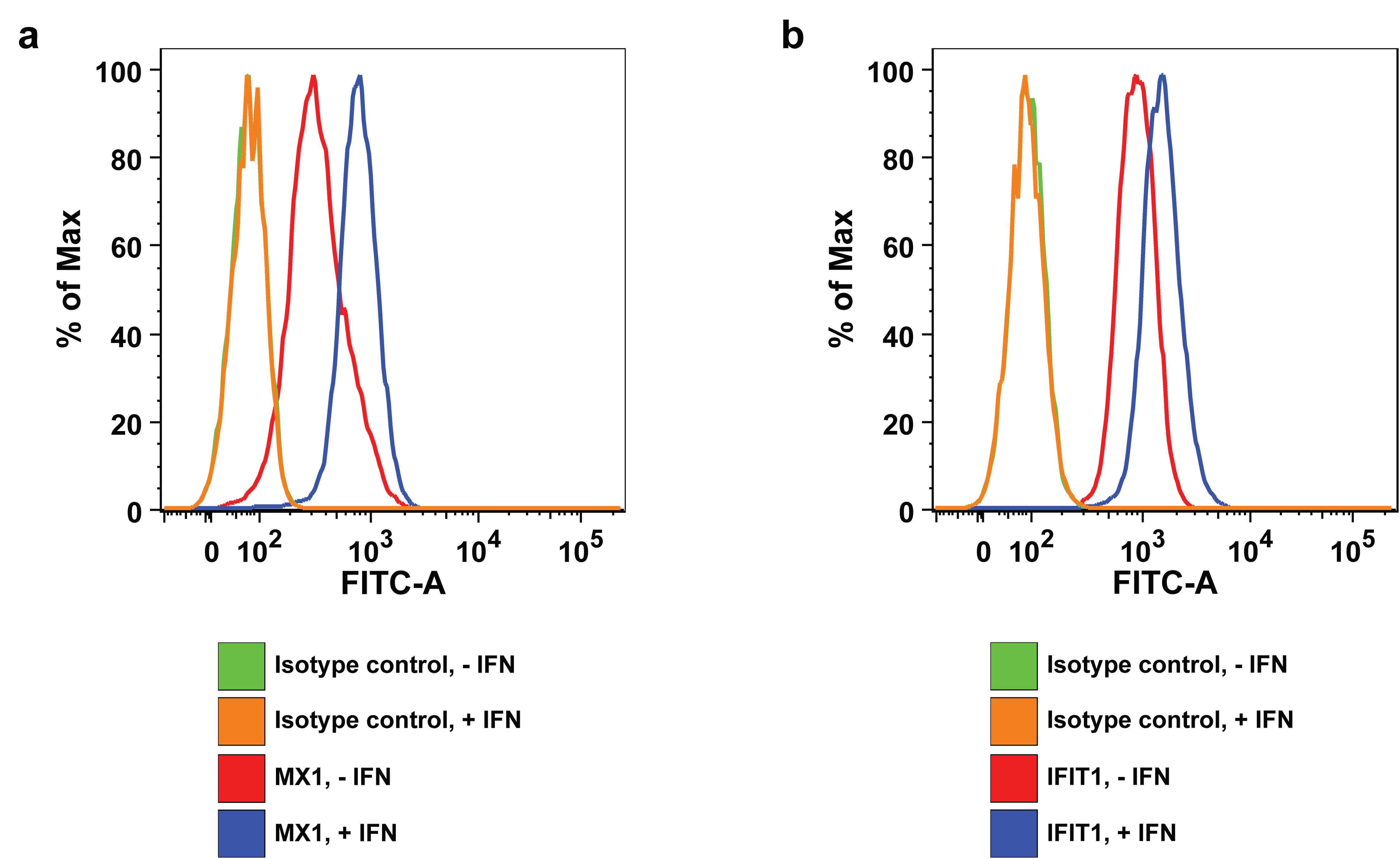

Supplement: S2 Fig — The immortalized pig-tailed macaque (Ptm) lymphocytes were left untreated or treated with 1000 U/ml of IFNα-2a for 24 hr. Cells were fixed, permeabilized and intracellular staining was performed for MX1 (a) and IFIT1 (b) followed by flow cytometric analysis. The histograms represent the expression of MX1 and IFIT1 as measured by fluorescein isothiocyanate (FITC)-conjugated secondary antibody in the untreated (red) or IFNα-2a-treated (blue) cells. Appropriate isotype control antibodies were used for intracellular staining of MX1 and IFIT1. The data are representative of two independent experiments. (TIF) [file ppat.1005727.s003.tif]

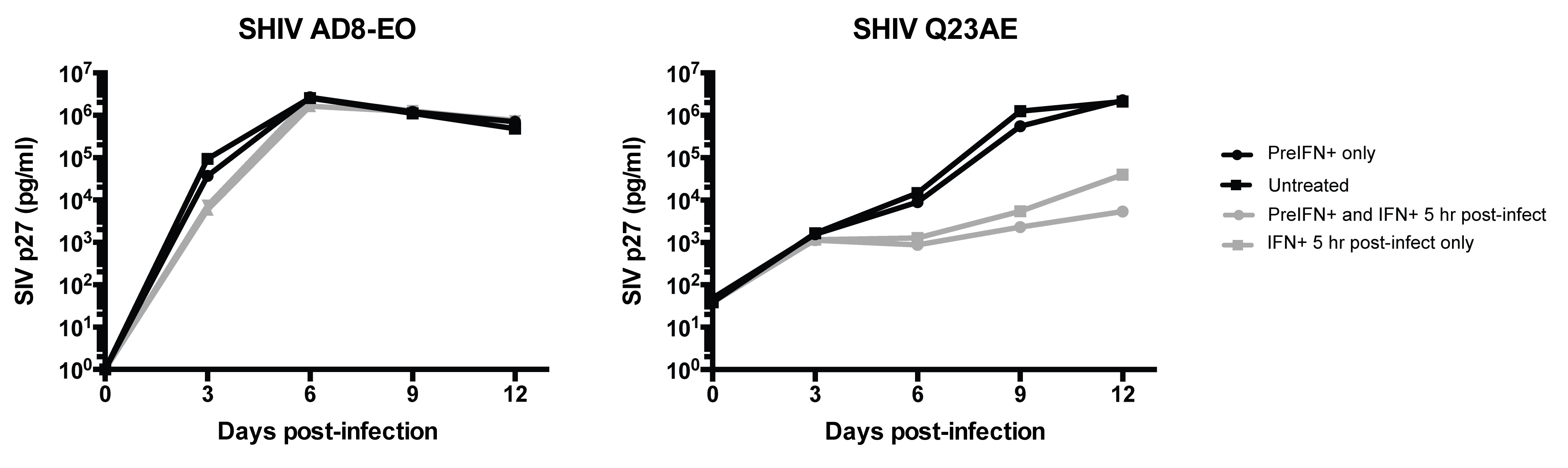

Supplement: S3 Fig — The ability of each SHIV to replicate in the presence of 1000 U/ml of IFNα-2a (gray lines) or absence of treatment (black lines) was assessed in immortalized pig-tailed macaque (Ptm) lymphocytes. Ptm cells were untreated (black square), only pre-treated 24 hr prior to infection with IFNα-2a (black circle), treated with IFNα-2a 5 hr post-infection (grey square), or both pre-treated and treated 5 hr post-infection with IFNα-2a (grey circle). The identity of each SHIV is indicated above the chart. SIV p27 concentration in infected cell supernatants is plotted vs. days post-infection. The data are representative of two independent experiments. (TIF) [file ppat.1005727.s004.tif]

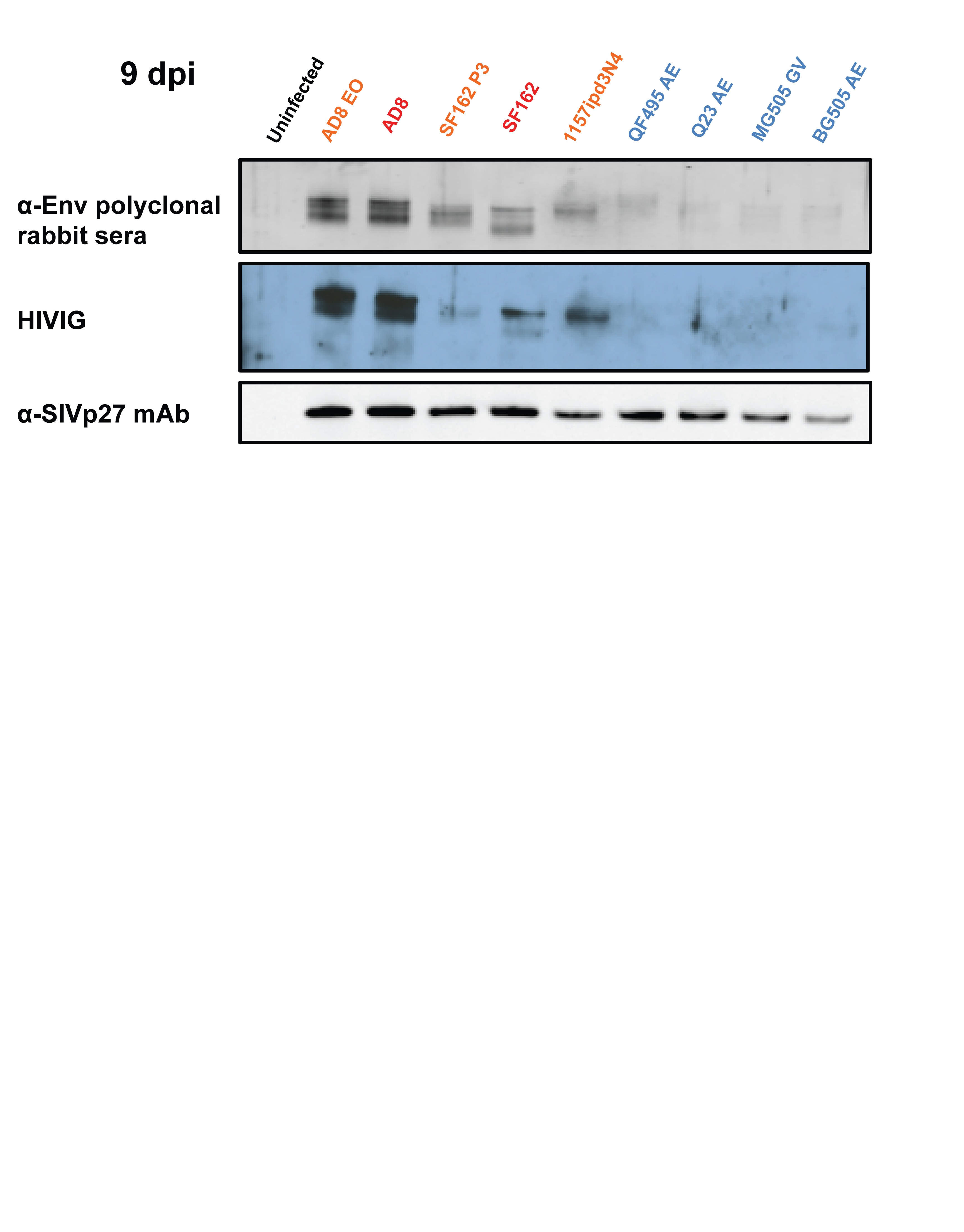

Supplement: S4 Fig — Five ng of SIV p27, as measured by ELISA, was loaded into each lane. The blot was probed with α-Env polyclonal rabbit sera [26] (top panel) and antibodies pooled from HIV-1+ patients (NIH AIDS Reagent Program) (bottom middle panel). The identity of the SHIV variant tested is indicated above each well, and SHIVs are color-coded as macaque-passaged (orange), lab-cultured (red) or circulating (blue) SHIVs. (TIF) [file ppat.1005727.s005.tif]

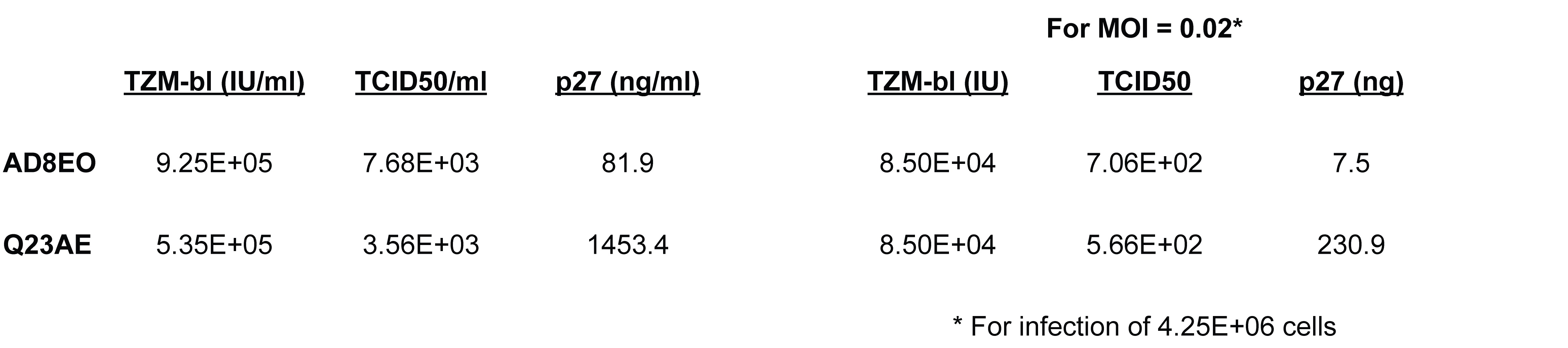

Supplement: S5 Fig — The number of infectious units (IU) per ml as determined by the TZM-bl assay and the viral titer as determined by TCID50 in immortalized Ptm lymphocytes are indicated. p27 represents the concentration of SIV p27 capsid protein in the viral stocks as determined by ELISA. At the right, the virus input to achieve an MOI of 0.02 for replication assays in macaque cells is indicated as number of infectious units or TCID50. In addition, the amount of SIV p27 added to the infections for each virus is indicated. (TIF) [file ppat.1005727.s006.tif]
